# Comparative visual performance of diffractive bifocal and rotationally asymmetric refractive intraocular lenses

**DOI:** 10.1038/s41598-022-24123-7

**Published:** 2022-11-12

**Authors:** Hirotaka Tanabe, Tomohiro Shojo, Tomofusa Yamauchi, Kosuke Takase, Masahiro Akada, Hitoshi Tabuchi

**Affiliations:** 1 Department of Ophthalmology, Tsukazaki Hospital, Himeji, Japan; 2 Department of Technology and Design Thinking for Medicine, Hiroshima University Graduate School of Biomedical and Health Sciences, Hiroshima, Japan

**Keywords:** Lens diseases, Eye diseases, Refractive errors

## Abstract

We compared the visual performance of a diffractive bifocal intraocular lens (IOL) with + 4.0 D near addition (ZMB00 [Johnson & Johnson Surgical Vision]) and a rotationally asymmetric refractive IOL with + 1.5 D near addition (LS-313 MF15 [Teleon Surgical BV]) 10 weeks after cataract patients’ last surgery for bilateral ZMB00 or LS-313 MF15 implantation between 2011 and 2020, with the lenses of each eye implanted within 3 months of each other. The ZMB00 and LS-313 MF15 groups comprised 1326 eyes of 663 patients (age: 67.0 ± 7.8 years; females/males, 518/145) and 448 eyes of 224 patients (73.6 ± 7.0 years; females/males, 125/99), respectively. A linear mixed-effects model using data for both eyes, with strict adjustments for sex, age, subjective refraction spherical equivalent, subjective refraction cylinder, corneal astigmatism, axial length, corneal higher-order aberrations, and pupil diameter, ensured statistical validity. Compared to LS-313 MF15, ZMB00 achieved significantly superior uncorrected near visual acuity, reduced higher-order aberrations (ocular/internal, scaled to a 4-mm pupil; Wavefront_4_post_Ocular_Total Higher-Order Aberration/Third/Fourth/Trefoil/Coma/Tetrafoil/Spherical, Wavefront_4_post_Internal_Astigmatism/Total Higher-Order Aberration/Third/Trefoil/Coma/Tetrafoil/Spherical), and superior distance and near spectacle independence (p < 0.00068, Wald test). Contrast sensitivity, measured without (visual angle of the test target: 6.3°/4.0°/2.5°/1.6°/1.0°/0.7°) or with glare (4.0°/2.5°/1.6°/1.0°/0.7°), was significantly better in the LS-313 MF15 than the ZMB00 group (p < 0.00068, Wald test).

## Introduction

To reduce spectacle dependence following cataract surgery, various types of intraocular lenses (IOLs) with multiple focal points or extended depth of focus (EDOF) have been developed. Because multifocal IOLs distribute incoming light to more than one focus, they can degrade contrast sensitivity and cause undesirable visual phenomena, including glare, halos, and starbursts^1–7^. In fact, many Japanese doctors and patients decide against multifocal IOLs, not only for financial reasons but also because of possible complications intrinsic to such IOLs. National surveys in 2019 and 2020 revealed that multifocal and EDOF IOLs, respectively, were used in only 3.9% and 5.1% of cataract surgeries^8,9^. However, technological innovations have been accumulating steadily for many years, such that patients who want to be completely free from spectacle use after cataract surgery may be able to attain this goal. Bifocal IOLs based on refractive or diffractive optics were developed in the late 1980s^10^, and several generations of technology later, the TECNIS ZMB00 diffractive bifocal IOL with + 4.0 diopters of near addition was introduced. This IOL provides excellent near visual acuity irrespective of pupil diameter, which depends on the amount of incoming light, because the overall diffractive structure of the IOL is characterized by TECNIS technology. To overcome the drawbacks of multifocal lenses, the TECNIS ZMB00 has negative spherical aberration (− 0.27 µm) to compensate for the positive corneal spherical aberration with a high Abbe's number of 55, which is a measure of the chromatic dispersion of a transparent material (change in refractive index by wavelength) with high values indicating low dispersion; the synergistic effect of reduced spherical aberration and reduced chromatic aberration is considered to improve the quality of vision^11,12^. Thus, the ZMB00 IOL has been one of the most popular diffractive bifocal IOLs in Japan for many years.

Regarding financial concerns, in March 2020, Japan’s advanced medical insurance services ceased to provide full financial support to insurance contractors through insurance companies for the use of registered multifocal and EDOF IOLs, causing many patients to encounter financial barriers to the use of these IOLs^13^. However, in 2019, the Lentis Comfort LS-313 MF15, a rotationally asymmetric refractive bifocal multifocal IOL with + 1.5 diopters of near addition, received an exceptional approval as the first non-monofocal lens supported by regular national medical insurance in Japan^13^. The Lentis Comfort LS-313 MF15 has a mild added power with a smooth transition area between the two different optical zones that helps suppress light scattering. Furthermore, its hydrophilic acrylic material with hydrophobic surface properties and a high Abbe's number of 57 is expected to enhance the quality of vision in several ways. Although it is not yet widely used in Japan (as mentioned above), its popularity is gradually increasing due to its high quality and affordability. Although bifocal IOLs such as the TECNIS ZMB00 provide good distance and near vision, results for intermediate vision are sometimes unsatisfactory^1,14,15^. Good intermediate vision, targeted by the Lentis Comfort IOL, is important for daily activities, especially given the ubiquitous use of computer tablets and smartphones.

To our knowledge, no studies have undertaken detailed comparative clinical analyses of these two IOLs on a large scale. Previously, we retrospectively compared the visual performance of two other IOLs (the TECNIS ZCB monofocal IOL and the TECNIS ZMB multifocal IOL) based on a large set of patient data^16^. A detailed statistical analysis successfully unveiled different characteristics of the two IOLs. In the present study, we analyzed data from a large number of patients at a single eye institute who received TECNIS ZMB00 multifocal IOLs and Lentis Comfort LS-313 MF15 IOLs between August 11, 2011, and March 26, 2020. We performed the same statistical analysis as in our previous report^16^ to clarify which product might be preferable for each patient who undergoes cataract surgery and for the health care system.

## Results

### Patient characteristics

The patient demographic variables and pre-/postoperative visual parameters used in the study are presented in Supplementary Table S1. The study included 1774 eyes of 887 patients: 1326 eyes of 663 patients in the ZMB00 IOL group (mean age: 67.0 ± 7.8 years; females/males, 518 [78.1%]/145 [21.9%]) and 448 eyes of 224 patients in the LS-313 MF15 IOL group (73.6 ± 7.0 years; females/males, 125 [55.8%]/99 [44.2%]).

### Comparison of postoperative parameters between the diffractive bifocal IOL with + 4.0 D near addition (TECNIS ZMB00 [Johnson & Johnson Surgical Vision] and the rotationally asymmetric refractive IOL with + 1.5 D near addition (Lentis Comfort LS-313 MF15 [Teleon Surgical BV])

**Figure 1:**
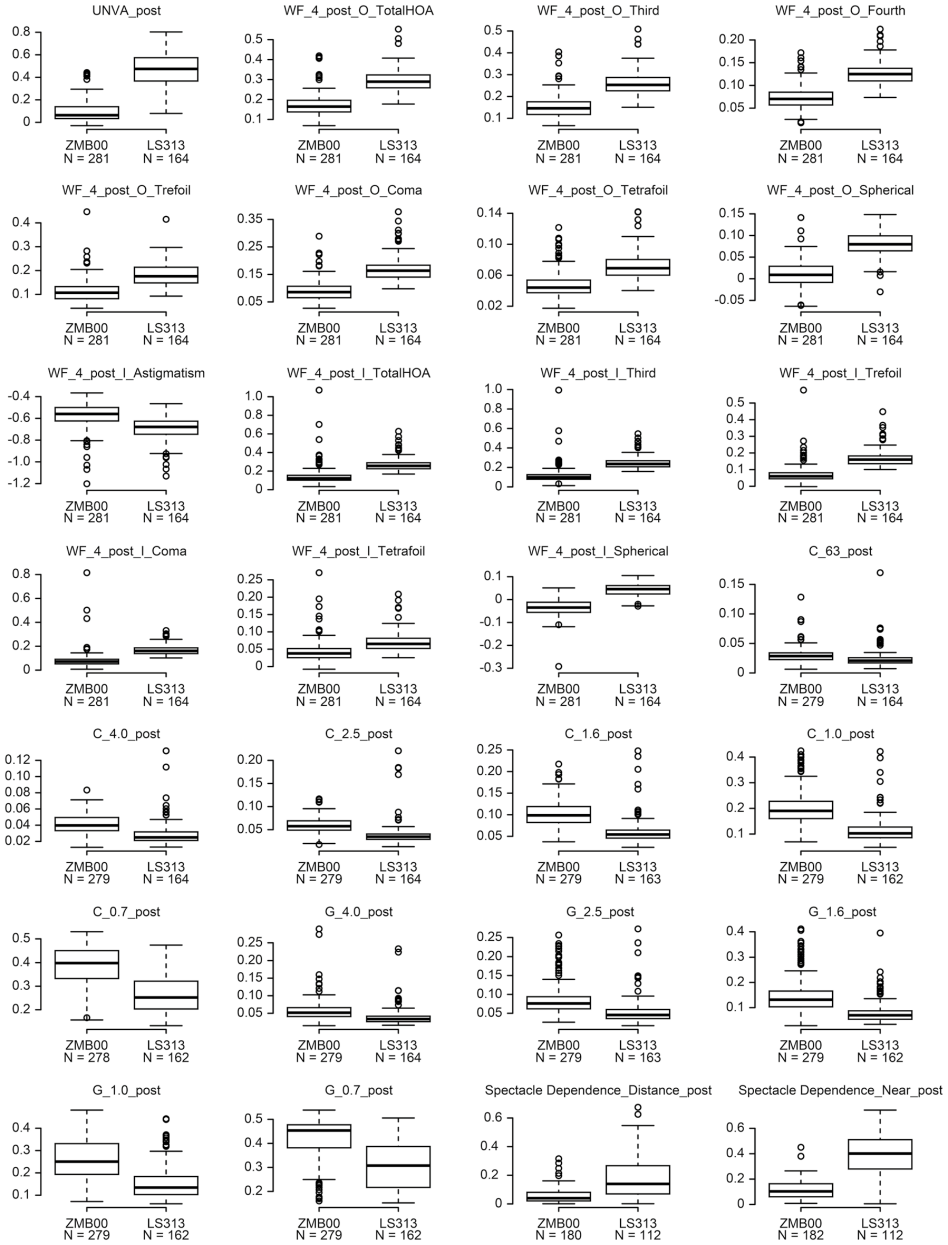
Parameters differing significantly between the diffractive bifocal (TECNIS ZMB00) and rotationally asymmetric refractive (Lentis Comfort LS-313 MF15) groups 10 weeks after surgery in both eyes. The line inside the box represents the median. To highlight outliers, the upper whisker is set to the maximum or the third quartile + 1.5 × IQR. The lower whisker indicates the minimum or the first quartile–1.5 × IQR. Each parameter was adjusted by multiple regression with the explanatory variables listed in Table 1. The two-sided Wald test was applied to evaluate the significance of differences between the two groups, and the significance level was set to 0.00068 using Bonferroni’s correction. UNVA: uncorrected near visual acuity; WF_4_post_O_: wavefront_4_post_ocular; WF_4_post_I_: wavefront_4_post_internal; HOA: higher-order aberration; C: contrast sensitivity; G: contrast sensitivity with glare.

Multiple regression analysis was conducted on all postoperative parameters of the two groups 10 weeks after surgery in both eyes in the same way as in our previous study^16^. The parameters were adjusted by multiple regression according to the explanatory variables in Table 1. The results of the analysis are shown in Supplementary Table S2. The ZMB00 group had significantly better uncorrected near visual acuity, smaller higher-order aberrations (ocular/internal, scaled to a pupil size of 4 mm) (Wavefront_4_post_Ocular [WF_4_post_O]_Total Higher-Order Aberration [TotalHOA]/Third/Fourth/Trefoil/Coma/Tetrafoil/Spherical, Wavefront_4_post_Internal [WF_4_post_I]_Astigmatism/TotalHOA/Third/Trefoil/Coma/Tetrafoil/Spherical), and better distance and near spectacle independence than the LS-313 MF15 group (p < 0.00068, Wald test) (Table 2 and Fig. 1). Additionally, the ZMB00 group had slightly but significantly better uncorrected/corrected distance visual acuity and smaller higher-order aberrations (ocular/internal, scaled to a pupil size of 4 mm) (WF_4_post_O_2ndAstig, WF_6_post_O_TotalHOA/Third/Fourth/Trefoil/Spherical, WF_6_post_I_Astigmatism/TotalHOA/Third/Fourth/Trefoil/Spherical) (p < 0.05, Wald test) (Table 2).

**Table 1:** Parameters in the diffractive bifocal (TECNIS ZMB00) and rotationally asymmetric refractive (Lentis Comfort LS-313 MF15) groups used to adjust the linear regression model: age, sex, axial length (at the time of surgery), subjective refraction spherical equivalent (SE), subjective refraction cylinder (CYL), corneal astigmatism (keratometric cylinder), corneal higher-order aberrations (astigmatism, total higher-order aberration (HOA), third, fourth, trefoil, coma, tetrafoil, second-order astigmatism (2ndAstig), spherical (scaled to a pupil size of 4 mm/6 mm), and pupil diameter (at 10 weeks after surgery).

(A) Categorical variable				
		N (%)		
Variable	Levels	ZMB00	LS-313 MF15	p value (Wald test)
Sex	F/M	518 (78.1)/145 (21.9)	125 (55.8)/99 (44.2)	1.739E−10

(B) Continuous variables				
	N, Mean ± SD			
Variable	ZMB00	LS313 MF15	p value (Wald test)	
Age	663, 67.043 ± 7.809	224, 73.647 ± 7.025	1.238E−55**	
SE	1069, 0.229 ± 0.448	368, − 0.224 ± 0.562	3.125E−42**	
CYL	849, − 0.785 ± 0.397	301, − 0.906 ± 0.514	2.079E−03*	
Corneal astigmatism	525, − 0.731 ± 0.413	420, − 0.813 ± 0.496	5.168E−02	
Axial length	1326, 24.045 ± 1.574	447, 23.731 ± 1.113	3.436E−02*	
**WF_4_post_C**				
Astigmatism	953, − 0.873 ± 0.501	323, − 0.954 ± 0.601	1.514E−01	
Total HOA	953, 0.204 ± 0.105	323, 0.225 ± 0.105	1.591E−04**	
Third	953, 0.174 ± 0.100	323, 0.199 ± 0.101	7.708E−06**	
Fourth	953, 0.098 ± 0.054	323, 0.096 ± 0.050	8.222E−01	
Trefoil	953, 0.131 ± 0.086	323, 0.150 ± 0.088	1.125E−04**	
Coma	953, 0.100 ± 0.075	323, 0.115 ± 0.079	8.695E−04**	
Tetrafoil	953, 0.059 ± 0.045	323, 0.057 ± 0.037	8.611E−01	
2ndAstig	953, 0.039 ± 0.030	323, 0.045 ± 0.031	4.168E−04**	
Spherical	953, 0.048 ± 0.047	323, 0.042 ± 0.049	1.927E−03**	
**WF_6_post_C**				
Astigmatism	868, − 0.623 ± 0.433	256, − 0.691 ± 0.468	6.351E−02	
Total HOA	868, 0.580 ± 0.435	256, 0.623 ± 0.350	4.407E−05**	
Third	868, 0.390 ± 0.310	256, 0.437 ± 0.262	5.672E−06**	
Fourth	868, 0.368 ± 0.249	256, 0.373 ± 0.194	2.257E−01	
Trefoil	868, 0.278 ± 0.236	256, 0.323 ± 0.231	2.021E−05**	
Coma	868, 0.245 ± 0.235	256, 0.258 ± 0.190	5.389E−02	
Tetrafoil	868, 0.169 ± 0.180	256, 0.156 ± 0.169	6.679E−02	
2ndAstig	868, 0.100 ± 0.158	256, 0.112 ± 0.113	3.131E−03*	
Spherical	868, 0.274 ± 0.162	256, 0.292 ± 0.118	7.630E−02	
Pupil diameter, post	980, 4.480 ± 0.871	334, 3.901 ± 0.797	1.244E−26**	

For categorical data, counts and frequencies are shown. Fisher’s exact test (two-sided) was used to compare categorical data for the diffractive bifocal and rotationally asymmetric refractive IOLs. For numerical data, the mean and standard deviation are shown, and the two-sided Mann–Whitney U test was used to compare numerical data for the diffractive bifocal and rotationally asymmetric refractive IOLs.

SE, subjective refraction spherical equivalent; CYL, subjective refraction cylinder; WF_4_post_C_, wavefront_4_post_corneal; HOA, higher-order aberration.

*p < 0.05, **p < 0.002 (= 0.05/25).

**Table 2:** Parameters demonstrating significant differences between the diffractive bifocal (TECNIS ZMB00) and rotationally asymmetric refractive (Lentis Comfort LS-313 MF15) groups 10 weeks after surgery in both eyes.

Response, post	After adjustment			
	ZMB00	LS-313 MF15	Coefficient (95% CI)	p value (Wald test)
UDVA	− 0.04 ± 0.07	0.04 ± 0.10	0.05 (0.02, 0.08)	3.047E−03*
CDVA	− 0.14 ± 0.04	− 0.09 ± 0.06	0.03 (0.01, 0.05)	1.143E−03*
UNVA	0.09 ± 0.09	0.46 ± 0.16	0.37 (0.32, 0.42)	6.512E−38**
**WF_4_post_O**				
TotalHOA	0.17 ± 0.05	0.29 ± 0.05	0.10 (0.08, 0.12)	3.030E− 17**
Third	0.15 ± 0.05	0.26 ± 0.05	0.09 (0.07, 0.11)	4.095E− 14**
Fourth	0.07 ± 0.02	0.13 ± 0.02	0.05 (0.04, 0.06)	6.791E− 19**
Trefoil	0.11 ± 0.04	0.18 ± 0.04	0.05 (0.04, 0.07)	1.986E− 09**
Coma	0.09 ± 0.03	0.17 ± 0.04	0.07 (0.05, 0.09)	6.104E− 11**
Tetrafoil	0.05 ± 0.02	0.07 ± 0.02	0.02 (0.01, 0.03)	6.471E− 07**
2^nd^Astig	0.04 ± 0.02	0.05 ± 0.02	0.01 (0.00, 0.01)	4.392E− 02*
Spherical	0.01 ± 0.03	0.08 ± 0.03	0.06 (0.05, 0.07)	2.822E−39**
**WF_4_post_I**				
Astigmatism	− 0.57 ± 0.11	− 0.70 ± 0.11	− 0.14 (− 0.22, − 0.06)	3.869E− 04**
TotalHOA	0.14 ± 0.08	0.27 ± 0.07	0.11 (0.09, 0.13)	2.662E− 22**
Third	0.11 ± 0.07	0.25 ± 0.06	0.11 (0.09, 0.13)	1.095E− 26**
Fourth	0.08 ± 0.04	0.10 ± 0.04	0.02 (0.01, 0.03)	4.680E− 03*
Trefoil	0.07 ± 0.05	0.17 ± 0.05	0.08 (0.07, 0.10)	9.993E− 34**
Coma	0.08 ± 0.06	0.17 ± 0.04	0.07 (0.06, 0.09)	3.536E− 14**
Tetrafoil	0.04 ± 0.03	0.07 ± 0.03	0.02 (0.01, 0.03)	1.741E−05**
Spherical	− 0.04 ± 0.03	0.04 ± 0.03	0.06 (0.05, 0.07)	2.822E−39**
**WF_6_post_O**				
TotalHOA	1.19 ± 0.87	2.28 ± 0.95	0.56 (0.21, 0.90)	1.773E−03*
Third	0.68 ± 0.41	1.21 ± 0.47	0.27 (0.03, 0.52)	3.191E−02*
Fourth	0.82 ± 0.73	1.56 ± 0.79	0.31 (0.07, 0.55)	1.199E−02*
Trefoil	0.47 ± 0.33	0.90 ± 0.35	0.27 (0.04, 0.50)	2.216E−02*
Spherical	0.39 ± 0.41	0.83 ± 0.39	0.19 (0.04, 0.34)	1.649E−02*
**WF_6_post_I**				
Astigmatism	− 0.94 ± 0.79	− 1.75 ± 0.82	− 0.36 (− 0.71, − 0.01)	4.263E−02*
TotalHOA	1.06 ± 0.86	2.13 ± 0.97	0.54 (0.19, 0.89)	2.610E−03*
Third	0.57 ± 0.44	1.13 ± 0.50	0.29 (0.04, 0.54)	2.488E−02*
Fourth	0.74 ± 0.67	1.39 ± 0.79	0.27 (0.02, 0.51)	3.261E−02*
Trefoil	0.35 ± 0.34	0.82 ± 0.36	0.28 (0.05, 0.51)	1.848E−02*
Spherical	0.11 ± 0.39	0.54 ± 0.36	0.19 (0.04, 0.34)	1.649E−02*
**Contrast sensitivity**				
C_6.3_post	0.03 ± 0.01	0.02 ± 0.01	− 0.01 (− 0.02, − 0.01)	2.316E−05**
C_4.0_post	0.04 ± 0.01	0.03 ± 0.02	− 0.02 (− 0.02, − 0.01)	2.313E−09**
C_2.5_post	0.06 ± 0.02	0.04 ± 0.03	− 0.03 (− 0.03, − 0.02)	4.356E−08**
C_1.6_post	0.10 ± 0.03	0.06 ± 0.03	− 0.05 (− 0.06, − 0.04)	1.484E−11**
C_1.0_post	0.20 ± 0.06	0.12 ± 0.05	− 0.11 (− 0.13, − 0.08)	4.075E−18**
C_0.7_post	0.39 ± 0.08	0.26 ± 0.08	− 0.15 (− 0.18, − 0.11)	9.348E−17**
**Contrast sensitivity with glare**				
G_6.3_post	0.04 ± 0.02	0.03 ± 0.01	− 0.01 (− 0.02, − 0.00)	2.413E− 02*
G_4.0_post	0.06 ± 0.03	0.04 ± 0.03	− 0.02 (− 0.03, − 0.01)	4.493E− 05**
G_2.5_post	0.08 ± 0.04	0.05 ± 0.03	− 0.03 (− 0.05, − 0.02)	9.900E− 06**
G_1.6_post	0.14 ± 0.06	0.08 ± 0.04	− 0.07 (− 0.09, − 0.05)	2.666E− 10**
G_1.0_post	0.27 ± 0.10	0.16 ± 0.08	− 0.13 (− 0.17, − 0.10)	3.401E− 14**
G_0.7_post	0.42 ± 0.08	0.30 ± 0.09	− 0.14 (− 0.17, − 0.10)	1.759E− 15**
**VFQ-25**				
Driving_General	81.75 ± 5.59	86.96 ± 6.62	5.45 (0.64, 10.25)	2.640E− 02*
Driving_Nighttime	40/27	8/33	0.94 (0.10, 1.77)	2.843E− 02*
**Spectacle dependence**				
Distance	90/0/0	50/0/6	2.12 (1.08, 3.17)	6.351E− 05**
Near	89/0/2	33/0/23	2.30 (1.58, 3.02)	3.678E− 10**

Each parameter was adjusted by multiple regression with the explanatory variables in Table 1. For each response variable, the mean and standard deviation for each numerical parameter or the counts for each categorical parameter (Spectacle Dependence: never/sometimes/always), the regression coefficient, its 95% confidence interval, and the p value (Wald test) are shown.

UDVA, uncorrected distance visual acuity; CDVA, corrected distance visual acuity; UNVA, uncorrected near visual acuity; WF_4_post_O, wavefront_4_post_ocular; WF_4_post_I: wavefront_4_post_internal; HOA, higher-order aberration.

*p < 0.05, **p < 0.00068.

**Figure 2:**
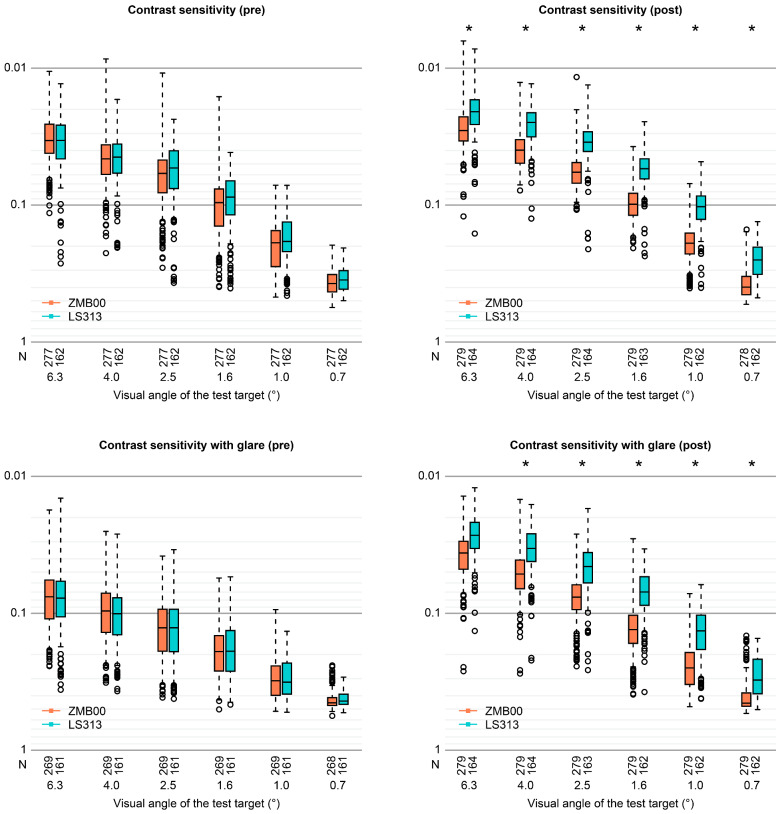
Contrast sensitivity with or without glare (visual angle of the test target: 6.3°/4.0°/2.5°/1.6°/1.0°/0.7°) in the diffractive bifocal (TECNIS ZMB00) and rotationally asymmetric refractive (Lentis Comfort LS-313 MF15) groups before and 10 weeks after surgery in both eyes. In the box-and-whisker plots, the bottom of the box indicates the first quartile, and the top of the box indicates the third quartile. The band inside the box represents the median. To highlight suspected outliers, the upper whisker indicates the maximum or the third quartile + 1.5 × IQR, while the lower whisker indicates the minimum or the first quartile-1.5 × IQR. Each parameter was adjusted by multiple linear regression with the explanatory variables in Table 1. The two-sided Wald test was applied to evaluate the significance of differences between the two groups, and the significance level was set to 0.0083 after Bonferroni’s correction.

**Figure 3:**
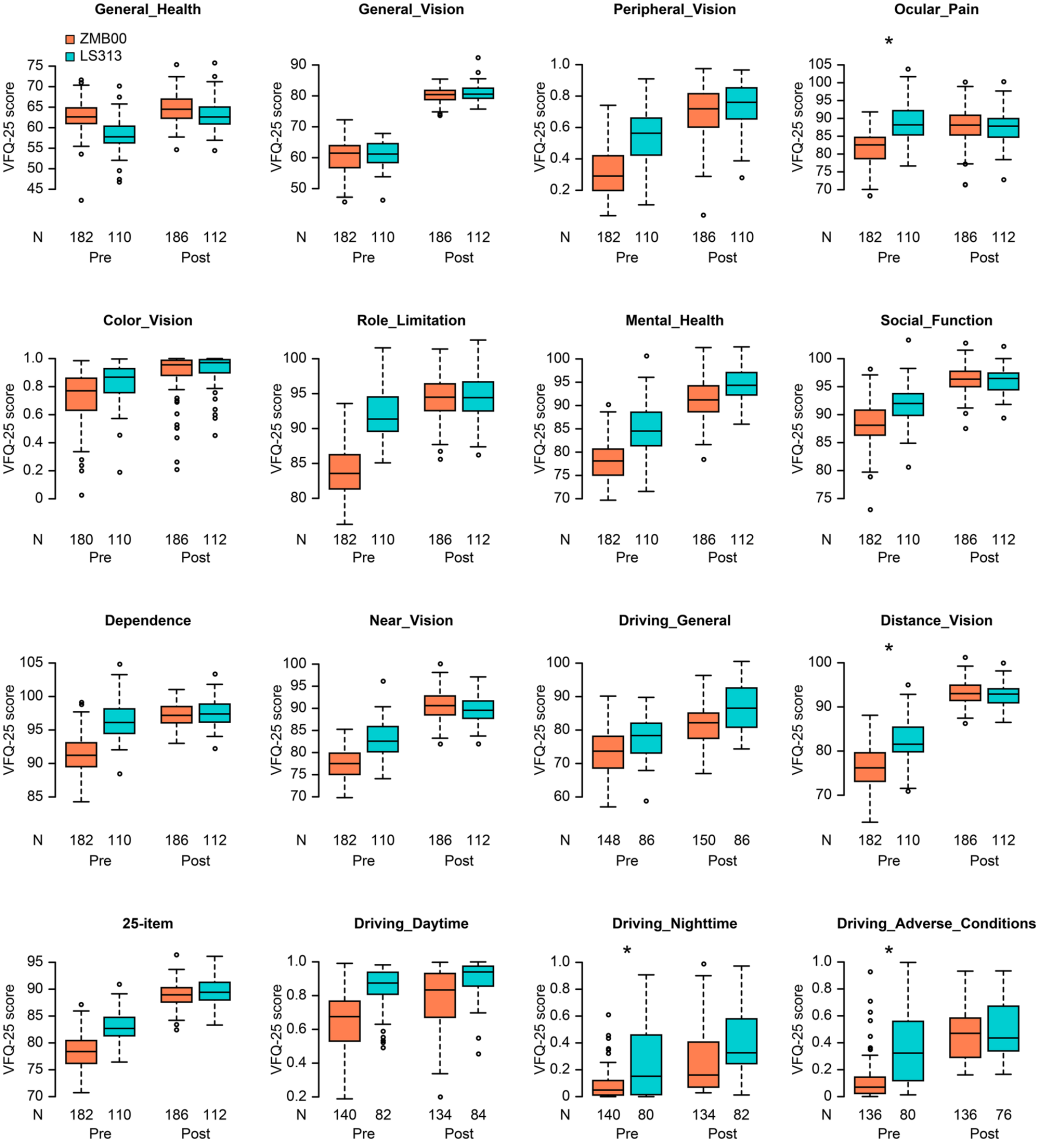
VFQ-25 scores in the diffractive bifocal (TECNIS ZMB00) and rotationally asymmetric refractive (Lentis Comfort LS-313 MF15) groups before and 10 weeks after surgery in both eyes. In the box-and-whisker plots, the bottom of the box indicates the first quartile, and the top of the box indicates the third quartile. The band inside the box represents the median. To highlight suspected outliers, the upper whisker indicates the maximum or the third quartile + 1.5 × IQR, while the lower whisker indicates the minimum or the first quartile-1.5 × IQR. Each parameter was adjusted by multiple linear regression with the explanatory variables in Table 1. The two-sided Wald test was applied to evaluate the significance of differences between the two groups, and the significance level was set to 0.003125 after Bonferroni’s correction. The asterisk * in this figure indicates a significant difference between the two groups satisfying p < 0.003125. In this figure, the predicted values for the following variables were the probabilities of whether the score was over 75 points: Peripheral_Vision, Color_Vision, Driving_Daytime, Driving_Nighttime, and Driving_Adverse_Conditions. Therefore, the y-axis scale of the figure for these variables is from 0 to 1.

Contrast sensitivity (visual angle of the test target: 6.3°/4.0°/2.5°/1.6°/1.0°/0.7°) and contrast sensitivity with glare (4.0°/2.5°/1.6°/1.0°/0.7°) were significantly better in the LS-313 MF15 IOL group (p < 0.00068, Wald test) (Table 2, Figs. 1 and 2). Contrast sensitivity with glare (6.3°) and the 25-item National Eye Institute Visual Function Questionnaire (NEI VFQ-25) scores for Driving_General/Nighttime were also slightly but significantly better in the LS-313 MF15 IOL group (p < 0.05, Wald test) (Table 2, Figs. 2 and 3).

### Correlation of postoperative parameters between the diffractive bifocal IOL with + 4.0 D near addition (TECNIS ZMB00 [Johnson & Johnson Surgical Vision] and the rotationally asymmetric refractive IOL with + 1.5 D near addition (Lentis Comfort LS-313 MF15 [Teleon Surgical BV])

**Figure 4:**
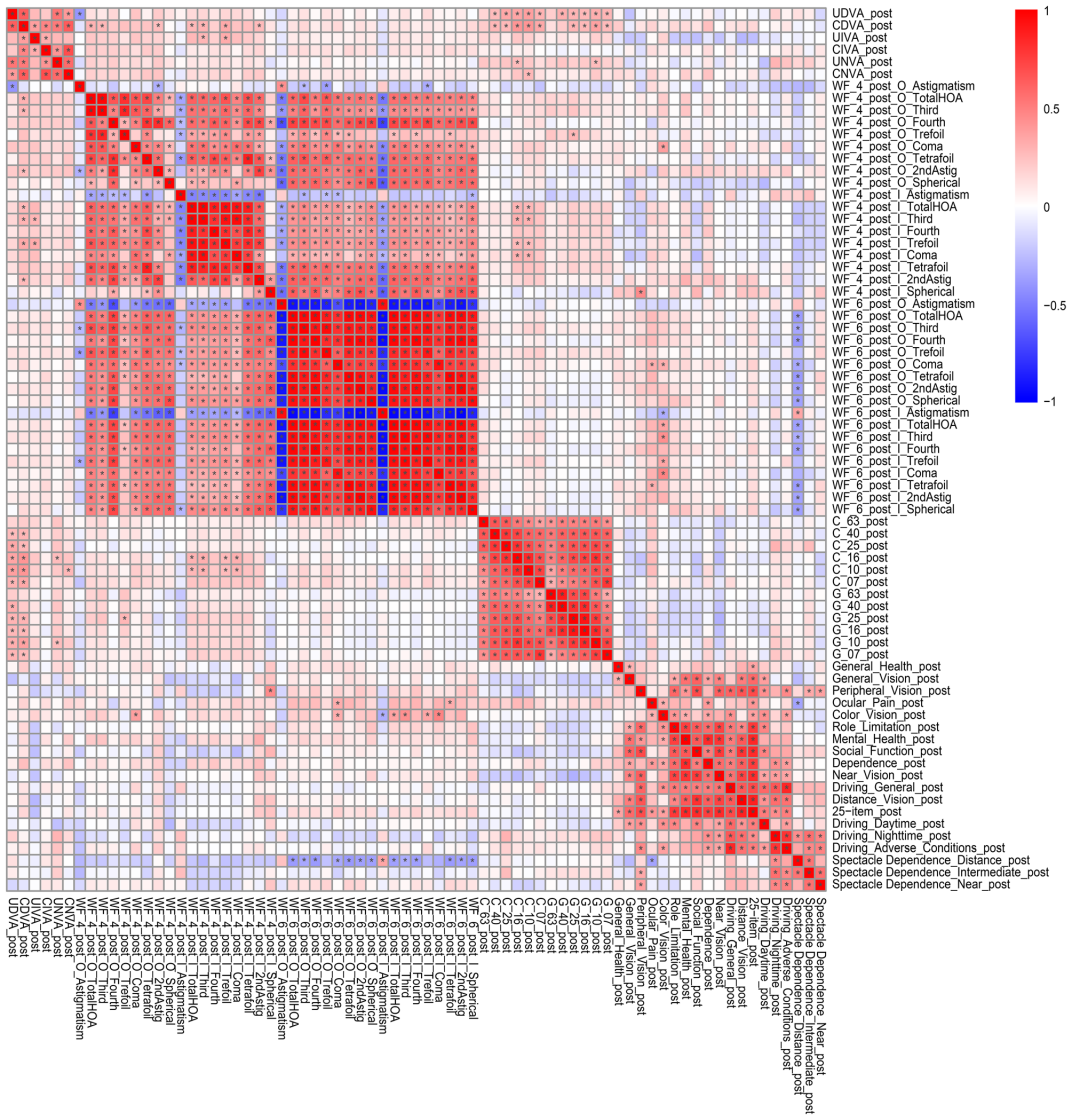
Heatmap of Pearson’s correlation coefficients between all possible combinations of variables, which were adjusted by multiple regression with the explanatory variables in Table 1, in the diffractive bifocal (TECNIS ZMB00) IOL group. The asterisk * in this figure indicates a significant correlation between two parameters at p < 0.00002 after Bonferroni’s correction. The two-sided t test was applied to evaluate the significance of differences between the two groups. The sample size for each parameter is shown in Supplementary Table S3(C). The illustration was performed using a commercially available software program (R, version 3.6.1; R Core Team, 2019, Vienna, Austria)^49^ (https://cran.r-project.org/web/packages/pheatmap/pheatmap.pdf).

**Figure 5:**
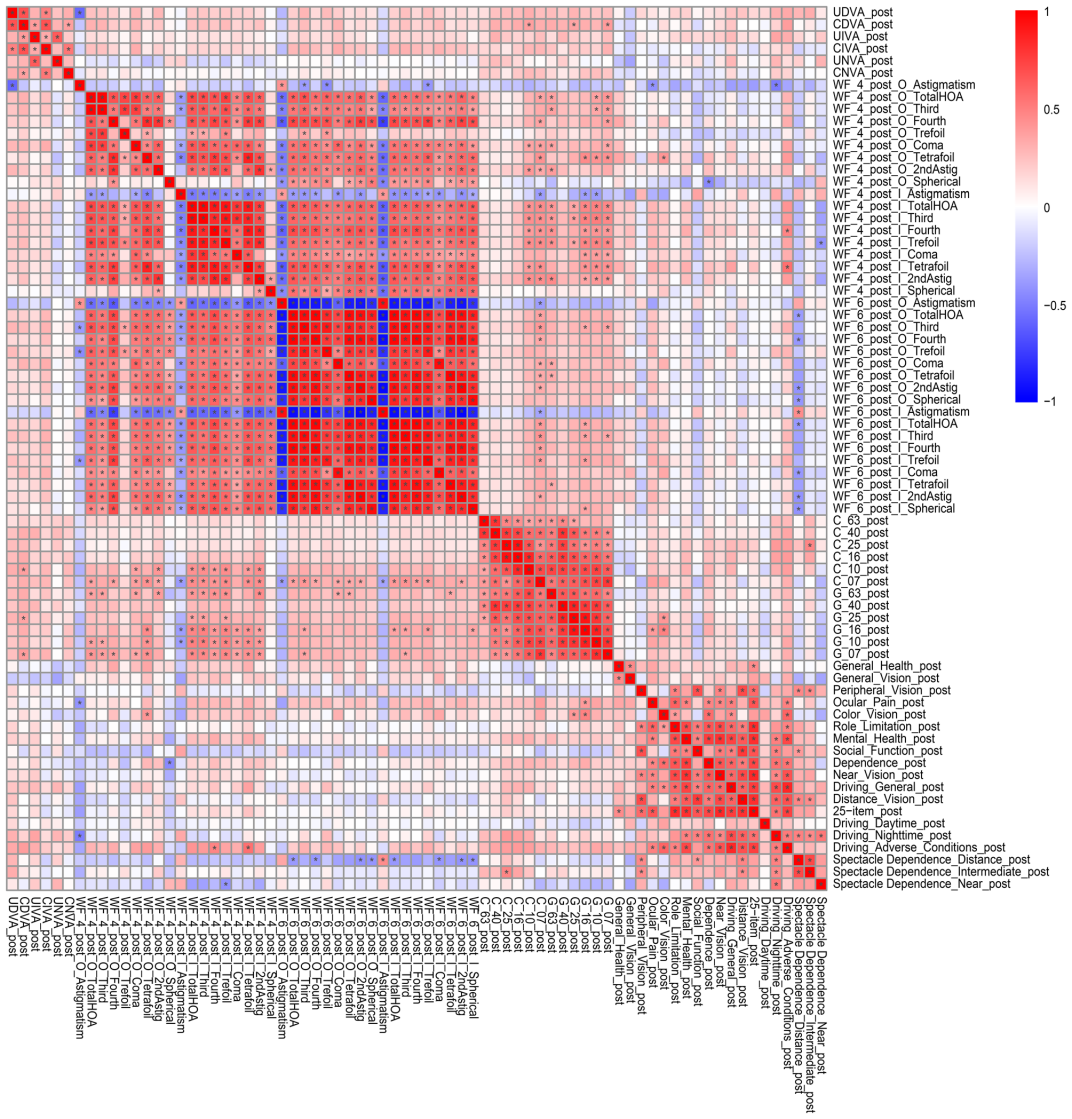
Heatmap of Pearson’s correlation coefficients between all possible combinations of variables, which were adjusted by multiple regression with the explanatory variables in Table 1, in the rotationally asymmetric refractive (Lentis Comfort LS-313 MF15) group. The asterisk * in this figure indicates a significant correlation between two parameters at p < 0.00002 after Bonferroni’s correction. The two-sided t test was applied to evaluate the significance of differences between the two groups. The sample size for each parameter is shown in Supplementary Table S4(C). The illustration was performed using a commercially available software program (R, version 3.6.1; R Core Team, 2019, Vienna, Austria)^49^ (https://cran.r-project.org/web/packages/pheatmap/pheatmap.pdf).

The correlation coefficients (A) and p values for the analyses (B) between all possible combinations of postoperative parameters for the two groups were adjusted by multiple regression using the explanatory variables in Table in the same way as in our previous study^16^; they are presented in Supplementary Table S3 and Fig. 4, and Supplementary Table S4 and Fig. 5, respectively.

## Discussion

In this study, we conducted a comprehensive clinical comparison of the visual performance of the TECNIS ZMB00 IOL and the Lentis Comfort LS-313 MF15 IOL in a large sample from a single eye institute. Although very high corrected visual acuity was maintained at all distances in both groups, corrected distance visual acuity was better in the ZMB00 group than in the LS-313 MF15 group (Table 2). Regarding uncorrected visual acuity, although uncorrected distance visual acuity (UDVA) was high in both IOL groups, the ZMB00 IOL group had significantly better uncorrected near visual acuity (UNVA) (Table 2 and Fig. 1) and UDVA (Table 2). On the other hand, UIVA was 0.20 logMAR in the ZMB00 group and 0.27 logMAR in the LS-313 MF15 group (Supplementary Table S2B), with no significant difference between the two groups. Oshika et al.^17^ showed that, over a 1-year study period, the Lentis Comfort LS-313 MF15 provided good distance and intermediate vision (approximately 0.0 logMAR UDVA and approximately 0.1 logMAR uncorrected intermediate visual acuity (UIVA) at 70 cm), whereas near visual acuity remained unsatisfactory (approximately 0.5 logMAR UNVA at 30 cm). The slightly worse UIVA of the LS-313 MF15 group in our study might be partly because intermediate VA was measured at 50 cm in this study. Because the Lentis Comfort LS-313 MF15 has + 1.5 D (+ 1.06 D on the corneal plane; 94 cm) of near addition power and ZMB00 has + 4.00 D (+ 3.2 D on the corneal plane; 31 cm) of near addition^16–21^, intermediate VA could be slightly worse at 50 cm than at 60–70 cm in the LS-313 MF15 group. This might also contribute to the lack of any significant difference between the UIVAs of the two groups. The distance at which intermediate visual acuity should be measured is a source of controversy, with opinions ranging from 50 to 100 cm^22^. The handheld devices and computers used in daily life commonly require users to rely on intermediate vision^23^. Within arm’s reach is also a practical distance for testing intermediate vision, with patients holding a test chart. By this standard, intermediate vision should be measured at a smaller distance in Japanese patients than in American or European patients due to differences between the populations in average body size and arm length. Therefore, we measured intermediate visual acuity at 50 cm, which was within the reach of most Japanese patients in this study.

We measured contrast sensitivity using the CGT-1000 instrument. This device can automatically measure contrast sensitivity^16,20,24^ at six sizes (visual angle of the test target: 6.3°/4.0°/2.5°/1.6°/1.0°/0.7°) and 13 contrast levels (0.01 to 0.64 contrast or 2.00 to 0.34 log_10_CS) with or without glare. In this study, the contrast sensitivity of the Lentis Comfort LS-313 MF15 group as measured with this device was significantly better than that of the ZMB00 group both with and without glare (Table 2, Figs. 1 and 2). Oshika et al.^17^ showed that the contrast sensitivity function of the Lentis Comfort LS-313 MF15 IOL group resembled that of an age-matched normal control sample over a 12-month study period after cataract surgery. Song et al.^21^ reported that the Lentis Comfort had a contrast sensitivity similar to those of monofocal IOLs and extended-range-of-vision IOLs (the Tecnis Symfony ZXR00). With the Tecnis Symphony ZXR00, the range of vision can be extended by elongating the focus based on an achromatic diffractive echelette design with nine diffractive rings, and this IOL was reported to have similar contrast sensitivity to monofocal IOLs^25^. On the other hand, we previously reported that the contrast sensitivity measured with the CGT-1000 was better in the monofocal group (ZCB00 IOL) than the multifocal group (ZMB00 IOL) at most frequencies both with and without glare^16^. In this study, the Lentis Comfort LS-313 MF15 IOL group had significantly better contrast sensitivity than the TECNIS ZMB00 IOL group with and without glare, which is consistent with the results of previous studies as a whole. Diffractive multifocal IOLs such as the TECNIS ZMB00 divide light into two foci; in general, TECNIS multifocal IOLs use 41% of incoming light for distance vision and 41% for near vision, regardless of the pupil diameter, while the remaining 18% is lost to higher-order scattering^26^. Recent research has confirmed that the use of 41% of light for distance vision allows photopic, high-contrast distance acuity comparable to that provided by monofocal IOLs^27–30^. The TECNIS ZMB00 IOL is a second-generation multifocal IOL with an aspherical design developed to improve contrast sensitivity by reducing or cancelling the normal positive spherical aberration of the cornea; the performance of this IOL has relatively little dependence on pupil size. Compared to spherical IOLs, aspherical IOLs reportedly show decreased wavefront spherical aberrations and improved contrast sensitivity^31–36^. Aspherical multifocal IOLs thus reduce the incidence and severity of halos and glare (i.e., steps at the edges of different ring zones), issues that are inherent to diffractive multifocal IOL designs and observed more often with spherical multifocal IOLs. In fact, rather than deterioration of contrast sensitivity with the use of diffractive multifocal IOLs, we have reported better contrast sensitivity both with and without glare in the multifocal group than in normal 60-year-old Japanese subjects assessed by Takahashi^16,20,37^. The Lentis Comfort LS-313 MF15, on the other hand, is a rotationally asymmetric, refractive IOL that combines a distance vision zone with a sector-shaped near vision zone featuring + 1.50 D power addition in the IOL plane (+ 1.06 D in the corneal plane)^17,21^, with a smooth transition between the two optical zones that divide light into two foci with an extended focus effect. Mild power addition with a smooth transition area between the two different optical zones helps suppress light scattering and undesirable phenomena. According to the manufacturer’s calculation, the Lentis Comfort has an optical loss of 5%, resulting in good postoperative contrast sensitivity, which is further enhanced by its hydrophilic structure. Previously, reports of calcification and opacification contributed to hydrophilic acrylic IOLs being less widely used than hydrophobic acrylic IOLs^38^. However, the latest generation of hydrophilic acrylic IOLs, such as the Lentis Comfort, avoid these problems; additional proposed advantages include reduced dysphotopsia, excellent biocompatibility, improved optical clarity, robustness, and protection against biocontamination^39^.

Higher-order aberrations (ocular/internal, scaled to a pupil size of 4 mm) (WF_4_post_O_TotalHOA/Third/Fourth/Trefoil/Coma/Tetrafoil/Spherical, WF_4_post_I_Astigmatism/TotalHOA/Third/Trefoil/Coma/Tetrafoil/Spherical) were significantly smaller in the ZMB00 IOL group (Table 2 and Fig. 1), and higher-order aberrations (ocular/internal, scaled to a pupil size of 4 mm) (WF_4_post_O_2ndAstig, WF_6_post_O_TotalHOA/Third/Fourth/Trefoil/Spherical, WF_6_post_I_Astigmatism/TotalHOA/Third/Fourth/Trefoil/Spherical) were less marked in the ZMB00 IOL group (Table 2). This might be partly because the anterior surface of the ZMB00 has negative spherical aberration (− 0.27 µm) as compensation for the positive spherical aberration of the cornea, whereas the Lentis Comfort IOL has an aspheric, aberration-free (0.0 μm) distance vision zone with a sector-shaped near vision zone^17,21^. Although aberrations can help improve the depth of focus, they can have a negative influence on contrast sensitivity^40,41^.

A heatmap of correlation coefficients revealed significant positive correlations between contrast sensitivity with/without glare (logarithm) and uncorrected/corrected distance visual acuity (logarithm) in the ZMB IOL group (Fig. 4). This implies that contrast sensitivity with/without glare may be critical for uncorrected/corrected distance visual acuity in this group. Additionally, significant positive correlations were observed between the higher-order aberrations (internal, scaled to a pupil size of 4 mm) and contrast sensitivity (*logarithm*) (Fig. 4); in other words, the higher-order aberrations had significantly negative correlations with contrast sensitivity. By contrast, the heatmap of correlation coefficients revealed significant positive correlations between contrast sensitivity with/without glare (*logarithm*) and higher-order aberrations (ocular/internal, scaled to a pupil size of 4 mm/6 mm) in the Lentis Comfort LS-313 MF15 IOL group (Fig. 5); in other words, contrast sensitivity correlated negatively with higher-order aberrations. Nochez et al.^40^ reported a significant negative correlation between total ocular spherical aberration and contrast sensitivity, and Piers et al.^41^ showed that contrast sensitivity was best when spherical aberration was completely corrected. The negative correlation observed between the higher-order aberrations and contrast sensitivity in both groups in our study appears consistent with previous reports.

In our study, approximately 82.2% of patients in the ZMB00 group were fully spectacle-independent after IOL implantation. This result is consistent with previous reports on ZMB00, in which 82.6% to 92.8% of patients achieved complete spectacle independence^14,27,42,43^. In contrast, the frequency of spectacle independence in our LS-313 MF15 group was approximately 39.5%. Distance/near spectacle independence was significantly higher in the ZMB00 group (Table 2 and Fig. 1). Multifocal glasses are widely used by Japanese people with presbyopia; patients who need spectacles for near vision often use multifocal glasses that correct distance vision as well. This may be a reason for the superior rate of distance spectacle dependence in the Lentis Comfort group, whose UDVA was as satisfactory as that of the ZMB00 group.

The NEI VFQ-25 is a self-report questionnaire used to measure vision-related health status^44,45^. This questionnaire can be used to evaluate changes in subjective visual function following cataract surgery, and it has been translated into several languages, including Japanese; the Japanese version was validated by Suzukamo et al.^46^ In our study, VFQ-25 scores for Driving_General/Nighttime were likely better in the LS-313 MF15 IOL group (Table 2 and Fig. 3). Difficulty in driving at night has been linked to perception of optical phenomena such as glare and halos^47^. Song et al.^21^ reported a significantly lower incidence of halos in the LS-313 MF15 group than in the Tecnis Symphony ZXR00 group, and visual quality scores at night were significantly better in the former group than in the monofocal group (implanted with the L-313, a lens made on the same platform as the LS-313 MF15). Yoo et al.^48^ reported that the Lentis Comfort LS-313 MF15 was associated with significantly lower rates of glare and halos than the same IOL with a basic design providing + 3 D near addition (Lentis M plus LS-313 MF30). Oshika et al.^17^ reported that the defocus curve of the Lentis Comfort presented a gradual decrease from distance vision to near vision, in contrast to the 2-peak curve seen with traditional bifocal IOLs. The low-addition design of the Lentis Comfort made it possible to have an elongated focal area and to minimize unfocused images that would generate halos. In this way, the incidence of undesirable photic phenomena might be reduced, which, in turn, might contribute to the high score for nighttime driving. Thus, our finding that the Lentis Comfort was associated with better nighttime driving scores than the ZMB00 appears to be consistent with prior studies.

One limitation of this study is that intermediate visual acuity was measured only at 50 cm and near visual acuity at 30 cm. In Japanese patients, visual acuity at these distances is essential for working at arm’s length and for reading. Ideally, however, visual acuity should be measured at a wider range of distances to gauge the performance of the lenses in detail. As mentioned above, evidence suggests that intermediate visual acuity should be measured at distances between 50 and 100 cm^22^. Distances within arm’s reach are practical for intermediate vision tests, for example, when the patient holds a test chart. As Japanese people are relatively short in average height and arm length, we measured intermediate visual acuity at 50 cm, assumed to be within the reach of most patients in the study.

A second limitation concerns the retrospective nature of the study; it is conceivable that the two patient groups had different social backgrounds. However, this large-scale single-center study followed a consistent protocol: after written informed consent was obtained from all the patients before surgery, we conducted the same series of pre- and postoperative examinations, including the VFQ-25, which captures information about the social background of the patients. We evaluated parameters 10 weeks after the last surgery in cataract patients who underwent bilateral ZMB00 or LS-313 MF15 implantation, with the right and left lenses implanted within 3 months of each other, and strictly adjusted for the variables of age, sex, axial length, subjective refraction SE, subjective refraction CYL, corneal astigmatism (keratometric cylinder), corneal higher-order aberrations, and pupil diameter. The data contain a mixture of items evaluated in both eyes together or in each eye separately. Our analysis accounted for any bias, as we used a linear mixed model and corrected for multiple observations for each eye per case. Although this study is retrospective, each patient receiving lenses was randomly and independently sampled, and all endpoints were measured. In statistical analysis, random assignment is widely assumed not to bias the outcomes of the analysis, even if the numbers of cases differ. An example of this is 1:n allocation in clinical trials.

In conclusion, we compared the visual performance of the TECNIS ZMB00 IOL and the Lentis Comfort LS-313 MF15 IOL. Patients in the ZMB00 group had better UNVA, smaller higher-order aberrations (ocular/internal, scaled to a pupil size of 4 mm) (WF_4_post_O_TotalHOA/Third/Fourth/Trefoil/Coma/Tetrafoil/Spherical, WF_4_post_I_Astigmatism/TotalHOA/Third/Trefoil/Coma/Tetrafoil/Spherical), and higher distance/near spectacle independence, whereas patients in the Lentis Comfort group had better contrast sensitivity (6.3°/4.0°/2.5°/1.6°/1.0°/0.7°) and contrast sensitivity with glare (4.0°/2.5°/1.6°/1.0°/0.7°). At a high performance level, the two IOL groups showed different characteristics with regard to various visual parameters.

## Methods

### Design

Retrospective comparative case series.

### Setting

Ophthalmology, Tsukazaki Hospital, Japan.

### Patients

We analyzed data from a consecutive series of cataract patients who underwent bilateral implantation of diffractive bifocal IOLs with + 4.0 D near addition (TECNIS ZMB00 [Johnson & Johnson Surgical Vision]) and rotationally asymmetric refractive IOLs with + 1.5 D near addition (Lentis Comfort LS-313 MF15 [Teleon Surgical] between August 11, 2011, and March 26, 2020, with the right and left lenses implanted within an interval of 3 months, as in our previous study^16^. Participants were recruited for enrollment in a consecutive case series study (outpatients with or without a doctor’s referral). There is no potential self-selection bias that might confound the results. The exclusion criteria were other ocular diseases that might affect visual function, |subjective equivalent (SE)|> 2.00 D, |subjective refraction cylinder (CYL)|> 3.00 D and |corneal astigmatism (keratometric cylinder)|> 3.00 D 10 weeks following surgery.

### Preoperative examination

Preoperative examinations were conducted as in our previous study^16^. All patients underwent full ophthalmologic examinations, including evaluations of the corneal curvature radius, corneal astigmatism, axial length, refractive status, ocular aberrations, pupil diameter, distance/intermediate/near visual acuity, contrast sensitivity, and contrast sensitivity under glare, as well as anterior segment evaluations using a slit lamp, tonometry and indirect fundoscopy. The quality of vision was evaluated using the Japanese version of the NEI VFQ-25^46^. The questionnaire was administered by experienced technicians or nurses in a face-to-face setting. Patients were also asked about their use of spectacles for distance, intermediate and near vision (the response options were “never”, “sometimes” and “always”).

Uncorrected (UDVA) and corrected distance visual acuity (CDVA) were both measured at 5.0 m. Uncorrected (UIVA) and corrected intermediate visual acuity (CIVA) were both measured at 0.5 m. Uncorrected (UNVA) and corrected near visual acuity (CNVA) were both measured at 0.3 m. Visual acuity was measured using the decimal visual acuity chart, with resulting decimal values converted to the logarithm of the minimum angle of resolution (logMAR) scale. The corneal curvature radius, corneal astigmatism and objective refractive status were measured using a KR-8900 autorefractor keratometer (Topcon, Tokyo, Japan). Axial length was measured using IOL Master (Carl Zeiss, Oberkochen, Germany) and AL-3000 (TOMEY, Nagoya, Japan) biometers. Contrast sensitivity and contrast sensitivity under glare (visual angle of the test target: 6.3°/4.0°/2.5°/1.6°/1.0°/0.7°; 13 contrast levels: 0.01–0.64 contrast or 2.00–0.34 log_10_CS) were measured using a CGT-1000 contrast glare tester (Takagi Seiko, Nakano, Japan)^16,20,24^, and pupil diameter and ocular aberrations were measured using a KR-1 W Wavefront Analyzer (Topcon, Tokyo, Japan). All measurements were taken by experienced technicians.

### IOLs and surgical techniques

Patients chose which IOLs to have implanted after receiving information about the advantages and disadvantages associated with each type as in our previous study^16^. Patients in the diffractive bifocal IOL group received TECNIS ZMB00, while those in the rotationally asymmetric refractive IOL group received Lentis Comfort LS-313 MF15 bilaterally. The goal for all eyes was emmetropia for distant vision.

The Tecnis® ZMB00 (Johnson & Johnson Surgical Vision, Santa Ana, CA, United States) is a single-piece, bifocal hydrophobic acrylic lens with a posterior diffractive surface and aspheric anterior surface that adds − 0.27 μm of spherical aberration to the human eye, presenting an addition of 4 D, corresponding to 3.2 D on the corneal plane^16,18–20^. This aspherical, modified prolate anterior surface designed to minimize spherical aberrations improves post-cataract surgery contrast sensitivity under mesopic conditions. It has additional bifocal diffraction gratings with + 4.0 diopters with clear acrylic optics measuring 6.0 mm in diameter.

The Lentis Comfort LS-313 MF15 (Teleon Surgical BV, Spankeren, Netherlands) is a foldable, single-piece, clear, UV-absorbing, plate-haptic IOL with an overall length of 11.0 mm and a 6.0 mm biconvex optic. It is made of hydrophilic acrylic material with hydrophobic surface properties. It is a rotationally asymmetric, refractive IOL combining an aspheric aberration-free (0.0 μm) distance vision zone with a sector-shaped near vision zone with a + 1.50 D add power on the IOL plane (+ 1.06 D on the corneal plane)^17,21^.

The cataract surgeries were performed by 18 experienced cataract surgeons using the same standard technique of sutureless microincision phacoemulsification and the same protocol. The surgical procedures consisted of topical anesthesia, the creation of a scleral or corneal incision of 1.8 to 2.8 mm, 5 mm of continuous capsulorhexis, phacoemulsification cataract extraction and IOL implantation with an injector.

### Postoperative examination

Patients were evaluated at 10 weeks after surgery using the same examination protocol that was used preoperatively.

### Statistical analyses

The sample size was calculated for an alpha of 0.00068 and a power of 0.80. A standard deviation in VA of 0.10 logMAR units was presumed, in addition to a minimum detectable difference of 1 line of VA (0.1 logMAR), based on our previous study^16^; this calculation suggested the inclusion of 39 eyes per group. The ZMB00 and LS-313 MF15 groups comprised 1326 eyes of 663 patients and 448 eyes of 224 patients, respectively, and thus the sample size was sufficient.

As in our previous study^16^, the two groups were compared on the following postoperative parameters 10 weeks after surgery in both eyes: (1) mixed-effects linear regression: visual acuity (uncorrected/corrected, distance/intermediate/near), contrast sensitivity (with/without glare), and higher-order aberrations (ocular/internal, scaled to a pupil size of 4 mm/6 mm); (2) linear regression model or logistic regression: VFQ-25 score; and (3) cumulative logistic regression: spectacle dependence (distance/intermediate/near). Both groups were adjusted for age, sex, axial length, subjective refraction spherical equivalent, subjective refraction cylinder, corneal astigmatism, corneal higher-order aberrations and pupil diameter. In regression analyses (2) and (3), the data were divided into two parts (left-eye data and right-eye data), and the regression model was applied to each dataset. Since discrete scores were observed for "Peripheral_Vision", "Color_Vision", "Driving_Daytime", "Driving_Nighttime", and "Driving_Adverse_Conditions" on the VFQ-25, we treated them as binary data. We divided the patients into two groups (those with scores of 75 or lower and those with scores above 75) and applied the logistic regression model to both groups. The threshold was determined from the distribution of the following variables: Peripheral_Vision, Color_Vision, Driving_Daytime, Driving_Nighttime, and Driving_Adverse_Conditions. A threshold of 75 was used in this study because most VFQ-25 scores are > 75 after surgery. The results of the left- and right-eye analyses were combined using the inverse variance method; the corrected values were calculated for the left- and right-eye datasets, and the average values were used.

In the regression analysis, the Wald test was applied to evaluate the significance of differences in postoperative parameters between the two groups, and the significance level was set at 0.00068 using Bonferroni’s correction. Correlations between postoperative parameters were calculated for the rotationally asymmetric refractive and diffractive bifocal groups, and a heatmap of Pearson’s correlation coefficients was generated for each group. In the correlation analysis, two-sided t-tests were used to evaluate whether the coefficient was significantly different from zero, and the significance level was set at 0.00002 after Bonferroni’s correction.

The statistical analyses were performed using a commercially available software program (R, version 3.6.1; R Core Team, 2019, Vienna, Austria)^49^.

### Ethics statement

This study conformed to the tenets of the Declaration of Helsinki and was approved by the Ethics Committee of Tsukazaki Hospital. All research was performed in accordance with relevant guidelines/regulations. Written informed consent was obtained from each subject. This study was registered as UMIN000035630: “Performance comparison among different intraocular lenses in cataract surgery”.

## Supplementary Information

Supplementary Legends.

Supplementary Table S1.

Supplementary Table S2.

Supplementary Table S3.

Supplementary Table S4.

## Data Availability

All data relevant to the study are included in this article or have been uploaded as supplementary information.
