# Supplementary material for: Comparative visual performance of diffractive bifocal and rotationally asymmetric refractive intraocular lenses
Source: Sci Rep. 2022 Nov 12;12:19394. doi: 10.1038/s41598-022-24123-7 (PMC9653499; doi:10.1038/s41598-022-24123-7)
Supplement: Supplementary file 1 — Supplementary Legends. [file 41598_2022_24123_MOESM1_ESM.docx]

**Supplementary Tables**

**Supplementary Table S1.** Patient demographics and pre-/postoperative visual parameters. For categorical data, each category and its count and frequency are shown; Fisher’s exact test (two-sided) was used to compare categorical data for the diffractive bifocal (TECNIS ZMB00) and rotationally asymmetric refractive (Lentis Comfort LS-313 MF15) IOLs. For numerical data, the mean and standard deviation are shown; a two-sided Mann–Whitney U test was used to compare numerical data for the bifocal and rotationally asymmetric refractive IOLs.

**Supplementary Table S2.** Results of multiple regression analyses of all postoperative parameters of the diffractive bifocal (TECNIS ZMB00) and rotationally asymmetric refractive (Lentis Comfort LS-313 MF15) groups 10 weeks after surgery in both eyes. For numerical parameters, multiple mixed linear regression or multiple linear regression was applied, and cumulative logistic regression was applied to the spectacle dependence parameters. In the multiple linear regression or cumulative logistic regression, the variables in Table 1 were used as the explanatory variables. For each response variable, the regression coefficient, its 95% confidence interval, and the p value (Wald test) are shown in (A). The original and corrected values (i.e., before and after adjustment with multiple linear regression) of the mean and standard deviation for each numerical parameter and the counts for each categorical parameter (Spectacle Dependence: never/sometimes/always), the regression coefficient, the 95% confidence interval, and the p value (Wald test) are shown in (B).

**Supplementary Table S3.** Pearson’s correlation coefficients (A) and p values from the correlation analysis performed with a two-sided t test (B) of all possible combinations of postoperative parameters, which were adjusted by multiple regression with the explanatory variables in Table 1, in the diffractive bifocal (TECNIS ZMB00) group. The sample size for calculating the correlation coefficients is shown in (C).

**Supplementary Table S4.** Pearson’s correlation coefficients (A) and p values from the correlation analysis conducted with two-sided t tests (B) of all possible combinations of postoperative parameters, adjusted by multiple regression with the explanatory variables in Table 1, in the rotationally asymmetric refractive (Lentis Comfort LS-313 MF15) group. The sample size for calculating the correlation coefficients is shown in (C).
